# ClinAgent: AI-assisted methodology for clinical trial data processing and statistical programming

**DOI:** 10.1093/biomethods/bpag032

**Published:** 2026-06-11

**Authors:** Jaime Yan

**Affiliations:** Department of Data Sciences, Harrisburg University of Science and Technology, Harrisburg, PA 17101, United States

**Keywords:** clinical trial methodology, artificial intelligence, statistical programming, Model Context Protocol, CDISC standards, automation, pandas (RRID: SCR_018214), SAS (RRID: SCR_008567)

## Abstract

Clinical trial statistical programming requires 12–24 full-time-equivalent months per Phase 3 study and remains a bottleneck in pharmaceutical research. Modern artificial intelligence coding agents reason capably but lack domain-specific tools: they cannot read proprietary statistical software datasets, parse analysis specifications, or generate standards-compliant code without extensive guidance. We present ClinAgent, a skill and tool layer that augments any artificial intelligence coding agent with clinical programming capabilities through Model Context Protocol tools. Its design separates minimal data access from rich domain logic: skills package prompts, rule engines, and decision trees encoding expert knowledge, while tools provide stateless input-output for statistical software datasets, spreadsheet specifications, and log files. In this single-study proof-of-concept evaluation, we validate ClinAgent’s nine skills on artifacts from a production Phase 2 cardiovascular study, with synthetic datasets spanning 13 analysis domains and 102 109 observations. All skills pass functional validation. On this small sample, deterministic components identify one error and seven warnings without false positives and match all 56 subject-level variables; corresponding confidence intervals are wide, so these point estimates should be read as upper bounds pending replication. Prompt-based specification generation, dependent on the underlying language model, reaches 72.1% derivation accuracy overall, above 96% in simple domains and below 55% in complex ones, indicating that generated specifications require expert review. Our contributions include an agent-augmentation architecture, nine validated skills, tool implementations for clinical data formats, and a validation methodology distinguishing deterministic tool correctness from language-model-dependent output.

## Introduction

### Clinical trial data processing as a bottleneck in disease research

Translating scientific discoveries into approved therapies requires rigorous clinical trials, yet the data processing and statistical programming that underpin these trials have become a critical bottleneck for research on human diseases. The pharmaceutical industry invests over $2.6 billion and 10–15 years to bring a single drug to market [1]. Within this process, clinical trial statistical programming [the conversion of raw clinical data into analysis datasets, tables, listings, and figures (TLFs)] is a critical path activity that directly impacts regulatory submission timelines and, consequently, patient access to new treatments. A typical Phase 3 clinical trial requires 8–15 Analysis Data Model (ADaM) domains, 100–500 TLFs, and 12–24 full-time-equivalent (FTE) months of programming effort [2, 3], with 30%–50% quality control overhead.

Despite significant advances in clinical data standards and statistical computing, the programming workflow remains largely manual, dependent on experienced Statistical Analysis System (SAS) programmers who are increasingly scarce and expensive. Protocol amendments, changes to finalized study designs, are a major driver of rework: 57% of protocols have at least one substantial amendment, with Phase III protocols averaging 2.3 amendments and median implementation costs of $535 000 per amendment [4]. A follow-up study found that amendment prevalence has since risen to 76% of protocols, with a mean of 3.3 amendments per protocol [5]. This bottleneck delays the availability of clinical evidence that informs treatment decisions for patients with cancer, cardiovascular disease, rare genetic disorders, and other conditions. Moreover, the data harmonization challenges encountered in clinical trials are mirrored across biological and biomedical research pipelines, including real-world data analytics and epidemiological studies. Automating parts of this pipeline could reduce the typical 12–24 FTE-month programming effort for Phase 3 studies.

### The rise of artificial intelligence coding agents

The advent of large language model (LLM) coding agents (including Augment Code, Cursor, GitHub Copilot [6], and Claude Code) has changed software engineering practice. These agents can understand natural language task descriptions, read and write files autonomously, execute code and interpret results, and iterate based on errors and feedback.

However, these general-purpose agents lack the domain-specific tools needed for clinical programming. They cannot read SAS7BDAT files, parse ADaM specifications, analyze SAS logs for regulatory-relevant issues, or generate CDISC-compliant code without extensive manual guidance. The SAS7BDAT file is the proprietary binary dataset format used by SAS software: each file stores variable metadata (names, labels, formats, types, lengths) together with row-oriented observations, and is the de facto transfer format between statistical programming and biostatistics teams. A typical Phase 3 study generates 50–150 such files across SDTM and ADaM domains; for example, the adsl.sas7bdat file produced in our validation study (Results section) contains 669 records by 56 variables, with subject identifier, treatment arm, and analysis-population flags. Without native readers for this format, off-the-shelf AI coding agents cannot inspect, validate, or compare such datasets. Recent work on AI methods in biomedical research has demonstrated the potential of domain-specific tooling to bridge this gap [7], yet no existing system provides integrated clinical programming support for AI agents.

### Research gap: the missing tool layer

The gap is not in AI reasoning capability. Modern agents can perform complex code reasoning. The gap is in domain-specific tooling: no clinical data tools (agents cannot natively read SAS7BDAT files, Excel specifications, or RTF outputs), no domain knowledge packaging (CDISC standards and derivation patterns are not accessible to agents), no workflow orchestration for the designed end-to-end clinical-programming pipeline, and no compliance infrastructure for Good Practice (GxP) audit trails.

AI has been applied to clinical trial design [7] and eligibility screening [8], yet statistical programming automation remains underexplored. Landman *et al.* [9] demonstrated the potential of generative AI for statistical analysis plan authoring, yet functionally tested designs for end-to-end regulatory-style programming workflows remain limited. No existing system simultaneously provides AI agent integration via standard protocols, an end-to-end workflow from specification to electronic submission (eSub), and tools for regulatory-style outputs demonstrated in a proof-of-concept evaluation.

### Our contribution: a skill and tool layer for clinical programming

We present ClinAgent, a skill and tool layer designed to augment any AI coding agent with clinical programming capabilities. ClinAgent is not an LLM or agent itself; rather, it is a collection of Skills (self-contained packages combining domain knowledge, embedded prompts, rule engines, and tool bindings), MCP Tools (Model Context Protocol [10] servers providing standardized data access), and Compliance Infrastructure (audit logging, data masking, and access control).

This design embodies the principle of “Thin MCP, Thick Skills”: data access tools are minimal and stateless, while domain logic is packaged into rich, testable skill definitions. Our contributions include:

1. Agent-Augmentation Architecture: A five-layer design enabling any AI coding agent to perform clinical programming without agent lock-in.
2. Nine Validated Skills: SK-001 (Study Setup) through SK-009 (eSub Packaging) covering the complete clinical programming workflow.
3. MCP Tool Suite: Six Model Context Protocol servers for clinical data operations.
4. Validation Framework: A proof-of-concept methodology for evaluating the software and functional capabilities of each skill independent of agent choice. Benchmarking across different language-model agents is intentionally out of scope (see Evaluation Methodology section and Limitation section); results in this article characterize the ClinAgent tooling itself, not the reasoning quality of any specific agent that invokes it.
5. Open Design: Architecture specifications enabling community extension.

### Article organization

Materials and methods section presents the CDISC background, related work, architecture design, skill library, and evaluation methodology. Results section reports validation results. Discussion section discusses implications, generalization beyond clinical trials, advantages over existing approaches, limitations, and future work.

## Materials and methods

### CDISC and analysis data standards: a primer

For readers unfamiliar with clinical trial data standards, this subsection provides essential context. Clinical trial data submitted to regulatory agencies (FDA, EMA, PMDA) must conform to standards developed by the Clinical Data Interchange Standards Consortium (CDISC). The Study Data Tabulation Model (SDTM) defines how raw clinical data are organized into domains such as Demographics (DM), Adverse Events (AE), and Laboratory (LB). The Analysis Data Model (ADaM) specifies how analysis-ready datasets are derived from SDTM, following implementation guides for subject-level datasets (ADSL), the Basic Data Structure (BDS), and occurrence datasets (OCCDS) [11]. Each ADaM dataset contains variables with specific derivation rules—some copied directly from SDTM, others requiring complex transformations (e.g. flag variables, derived timing variables, and parameter-driven structures). Tables, Listings, and Figures (TLFs) are the statistical outputs generated from ADaM datasets for clinical study reports. The entire workflow proceeds as: Protocol → SDTM → ADaM → TLFs → eSub Package. Each stage is subject to Good Clinical Practice requirements. ClinAgent operates within this framework, providing tools that automate portions of the ADaM-through-eSub pipeline.

### Clinical trial statistical programming workflow

Clinical trial statistical programming transforms raw clinical data into regulatory-compliant analysis datasets and outputs following CDISC standards. The typical workflow requires specification development, code writing, execution, quality control, and validation, all subject to GxP requirements. Modern programming teams use SAS as the primary language, with organization-specific macro libraries providing standardized analytical procedures.

### Related work

#### LLM agent architectures

Recent advances in LLM-based agents have produced several architectural patterns. ReAct (Reasoning + Acting) [12] interleaves reasoning traces with tool use. Tool-augmented LLMs such as Toolformer [13] enable LLMs to invoke external tools. Multi-agent systems like AutoGen [14] orchestrate multiple specialized agents. The Model Context Protocol (MCP) [10] is an open protocol standardizing how LLMs connect to data sources and tools through JSON–RPC interfaces.

#### AI in clinical research

AI applications in clinical research have primarily focused on trial design [7], data management, medical coding, and document generation. Recent work includes LLM-based trial matching [8], CDISC-compliant specification tools [15], cross-study analysis packages [16], multi-agent knowledge engines [17], and regulatory report generation. The CDISC Analysis Results Standard (ARS) [18] provides a metadata-driven approach to TLF specification but requires implementation tooling.

#### Gap analysis

Table 1 compares existing approaches with our framework.

**Table 1:** Comparison of AI approaches for clinical programming^a^.

Capability	Copilot	AutoGen	Rule macros	ClinAgent
Code completion	✓	✓	–	✓
Workflow automation	–	Partial	–	✓
CDISC knowledge	–	–	Partial	✓
GxP compliance	–	–	✓	✓
Quality control	–	–	Partial	✓
LLM-agnostic	–	✓	NA	✓
End-to-end workflow design	–	–	–	✓

a ✓ = supported; partial = limited support; – = not supported; NA = not applicable.

No existing system provides AI agent integration via standard protocols, end-to-end workflow, and validated tools for regulatory-style outputs. ClinAgent addresses this gap.

### Architecture design

#### Core concept: agent augmentation

ClinAgent is not an AI agent or LLM system. It is a tool and skill layer that augments existing AI coding agents with clinical programming capabilities. Table 2 contrasts this approach with traditional agent frameworks.

**Table 2:** ClinAgent versus traditional agent frameworks.

Aspect	Traditional frameworks	ClinAgent
LLM integration	Built-in, specific model	None: uses user’s agent
Agent logic	Framework provides	User’s agent provides
Domain tools	Generic	Clinical-specific
Prompt engineering	User responsibility	Packaged in skills
Portability	Tied to framework	Works with any MCP agent

#### Capability gap

Table 3 compares what clinical programmers can accomplish with generic AI agents versus ClinAgent-augmented agents.

**Table 3:** Capability comparison: generic AI agents versus ClinAgent-augmented agents^a^

Clinical programming task	Copilot	Claude	Cursor	Augment	+ClinAgent
Read SAS7BDAT datasets natively	✗	✗	✗	✗	✓
Parse ADaM specification Excel	✗	✗	✗	✗	✓
Classify SAS log issues (ERROR/WARN)	△	△	△	△	✓
Generate CDISC-compliant code	△	△	△	△	✓
Validate ADaM datasets against spec	✗	✗	✗	✗	✓
Compare RTF outputs programmatically	✗	✗	✗	✗	✓
Apply company-specific macro catalog	✗	✗	✗	✗	✓
Create standardized eSub packages	✗	✗	✗	✗	✓
GxP-aligned audit logging	✗	✗	✗	✗	✓
PHI/PII data masking in context	✗	✗	✗	✗	✓

a ✓= native capability, △ = possible with extensive prompting, ✗ = not possible.

#### Design principles

Principle 1: Bring Your Own Agent. Users choose their preferred AI coding agent. ClinAgent provides domain-specific tools.

Principle 2: Thin MCP, Thick Skills. The MCP layer provides minimal, stateless data operations. All domain logic resides in Skills, enabling independent testing, easy updates, and reusable tools.

Principle 3: Prompts as Packaged Expertise. Skills contain pre-built prompts, examples, and constraints. The agent receives domain guidance automatically.

Principle 4: Deterministic Where It Matters. Error classification, validation rules, and compliance checks are encoded as rule engines, not LLM calls.

#### Five-layer architecture

**Figure 1:**
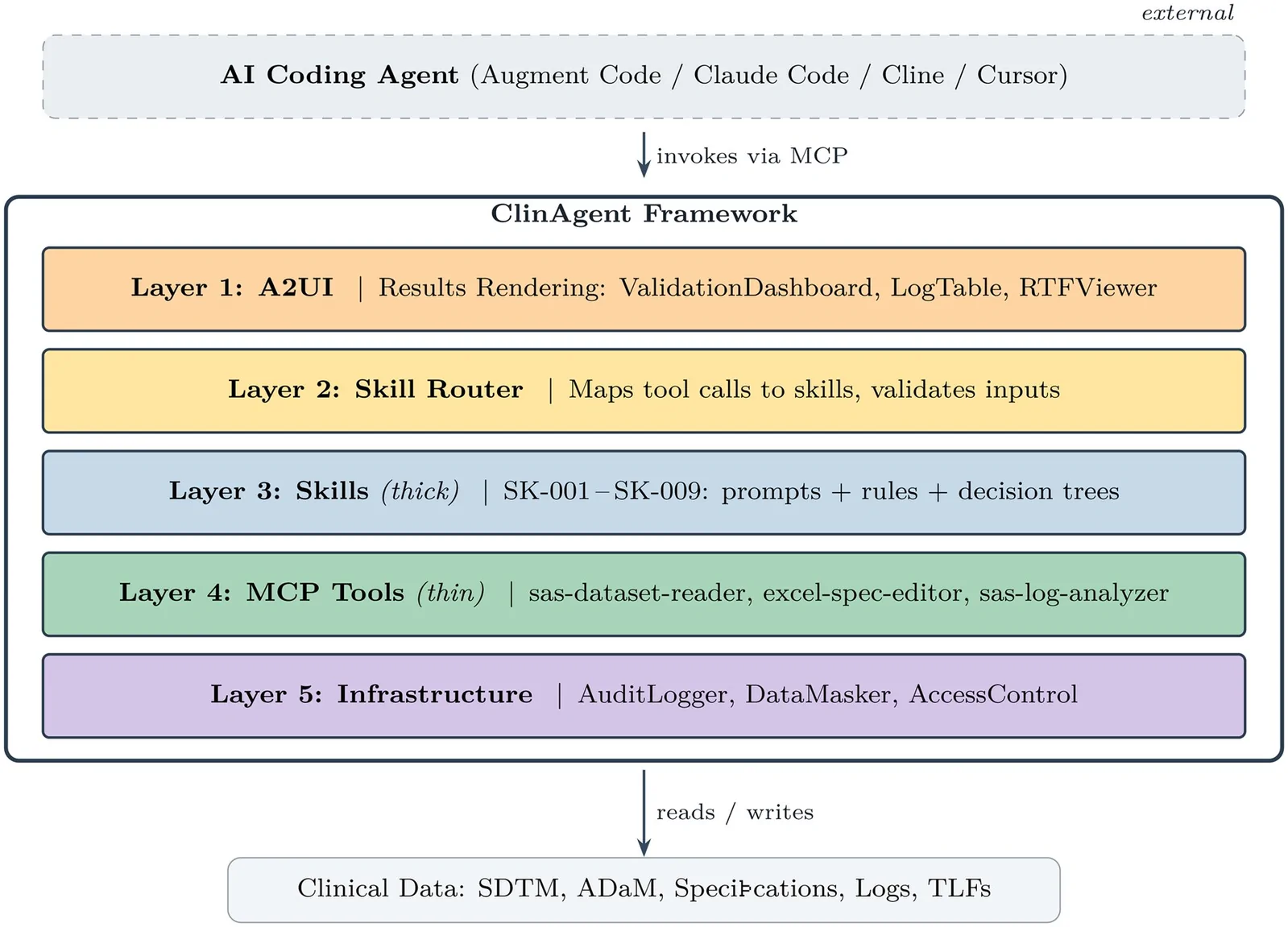
ClinAgent architecture. The framework provides skills and MCP tools that any AI coding agent can invoke. The agent handles reasoning; ClinAgent provides domain expertise and data access. Five layers are shown: A2UI (results rendering), skill router, skills (the “thick” layer containing domain logic), MCP tools (the “thin” layer providing stateless data access), and infrastructure (compliance components including AuditLogger, DataMasker, and AccessControl). external agents connect via the model context protocol.

Figure 1 shows how ClinAgent layers interact with external agents.

#### Layer details

External: AI Coding Agent. The agent is not part of ClinAgent. Users work with their preferred agent (Augment Code, Claude Code, Cline, Cursor). These agents connect to ClinAgent via MCP.

Layer 3: Skills (The “Thick” Layer). Each skill is a self-contained package combining domain knowledge (CDISC standards, SAS patterns), embedded prompts, rule engines, and tool bindings. When an agent invokes a skill, it receives the prompt template, access to MCP tools, and the rule engine for deterministic classification.

Listing 1: Skill Structure (SK-005 Log Reviewer)

1 {

2  ”skill_id”: “SK-005”,

3  ”name”: “log-reviewer”,

4  ”prompt_template”: {

5  ”system”: “You are a SAS log analyst…”,

6  ”few_shot_examples”: […],

7  ”constraints”: [“Never ignore ERROR lines”]

8  },

9  ”rule_engine”: {

10  ”error_patterns”: [“^ERROR:”, “^ERROR [0-9]+-”],

11  ”warning_patterns”: [“^WARNING:”],

12  ”false_positive_warnings”: [“Unable to copy SASUSER”]

13  },

14  ”mcp_tools_required”: [“sas-log-analyzer.read_log”]

15 }

Layer 4: MCP Tools (The “Thin” Layer). MCP servers provide stateless data operations containing no domain logic. They do not know what ADSL means or which variables matter. That knowledge lives in Skills.

Layer 5: Infrastructure (Compliance). Provides infrastructure features aligned with regulatory requirements: AuditLogger records every tool invocation; DataMasker removes protected health information (PHI) and personally identifiable information (PII) before data reaches agent context; AccessControl enforces role-based permissions; ContextMinimizer sends only necessary data. These features are design enablers; formal CSV and qualification within a specific adopter’s quality management system remain the responsibility of the adopting organization (see Regulatory Compliance section).

#### Concrete workflow walkthrough

**Figure 2:**
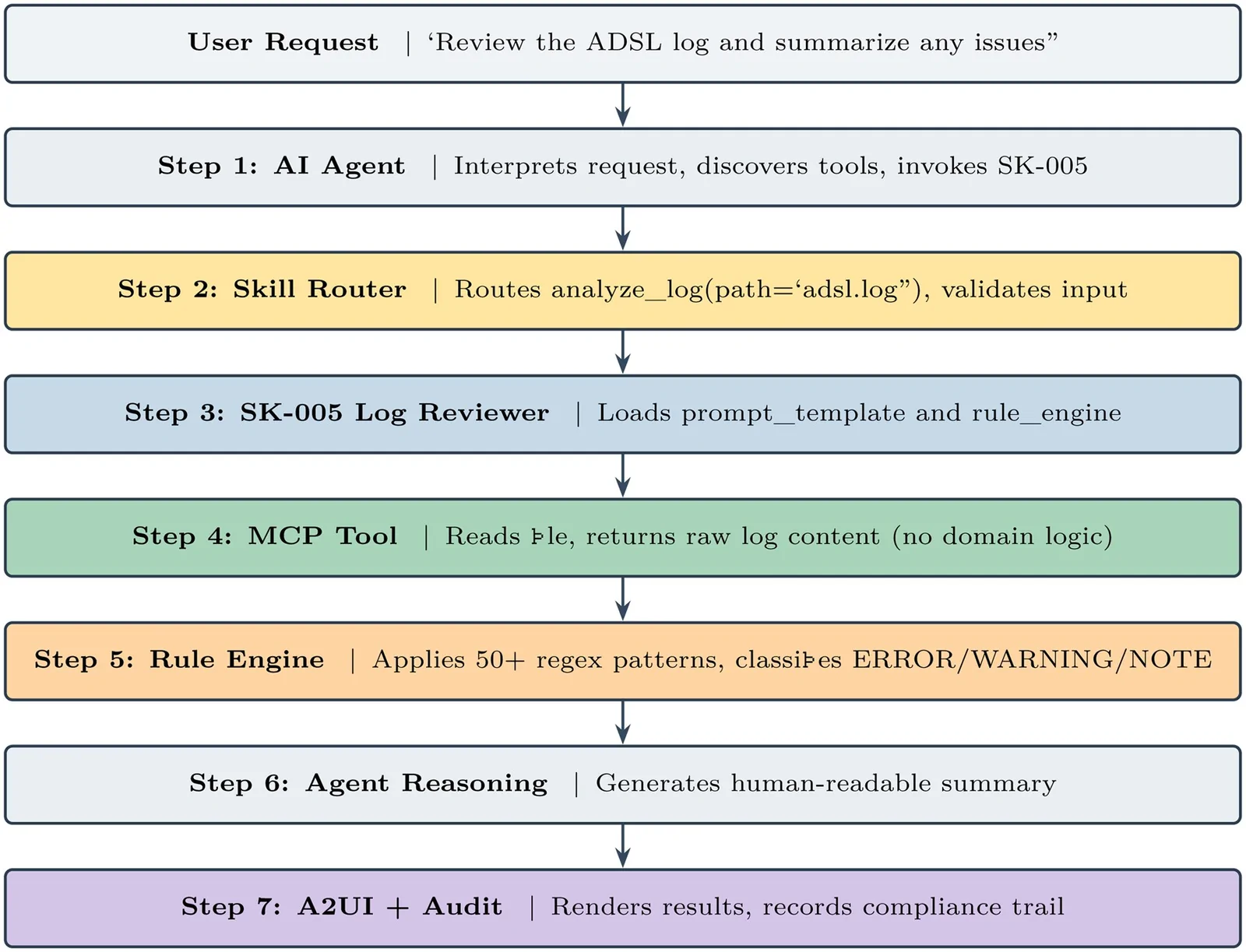
Complete workflow trace: log review request. The flow shows how a user request (“review the ADSL log and summarize any issues”) passes through the ClinAgent framework layers: the AI agent interprets the request, the skill router validates and routes, SK-005 loads domain prompts and rules, the MCP tool reads the file, the rule engine applies 50+ regex patterns for deterministic classification, the agent generates a human-readable summary, and A2UI renders results with audit logging.

Figure 2 traces a complete interaction for a log review request.

### Skill library

#### Skill catalog overview

ClinAgent defines nine validated skill packages covering the complete clinical programming workflow (Table 4).

**Table 4:** ClinAgent skill catalog

ID	Name	Purpose	MCP tools
SK-001	StudySetup	Create folder structure	filesystem
SK-002	SpecEditor	Edit ADaM specifications	excel-spec-editor
SK-003	ADaMCoder	Generate ADaM programs	excel-spec, filesystem
SK-004	SASRunner	Execute SAS programs	process, filesystem
SK-005	LogReviewer	Analyze SAS logs	sas-log-analyzer
SK-006	DataValidator	Validate datasets vs spec	sas-dataset-reader
SK-007	TLFCoder	Generate TLF programs	macro-catalog
SK-008	RTFQC	Compare RTF outputs	rtf-comparator
SK-009	eSubPackager	Create eSub package	filesystem

**Table 5:** Test study artifact characteristics

Characteristic	Count	Details
ADaM domains	13	ADSL, ADAE, ADBASE, ADCM, ADDILI, ADEFF, ADEG, ADEX, ADFAS, ADLB, ADMH, ADSOC, ADVS
Synthetic observations	102 109	Generated using Python Faker library (STUDY-A; proprietary synthetic data)
Total variables	764	Across all domains (STUDY-A)
TLF outputs	20	12 Tables, 3 listings, 5 figures
TLF programs	23	16 Within ClinAgent scope (7 legacy/exploratory excluded)
ADaM programs	10	

#### Rule engines

Skills encode domain expertise in JSON rule engines. Example from SK-005:

Listing 2: Error Classification Rules

1 {

2  ”error_patterns”: [

3  {

4  ”pattern”: “ERROR: Variable .+ not found”,

5  ”category”: “variable_reference”,

6  ”severity”: “ERROR”,

7  ”suggestion”: “Check variable name spelling and scope”

8  },

9  {

10  ”pattern”: “WARNING: Variable .+ is uninitialized”,

11  ”category”: “uninitialized_var”,

12  ”severity”: “WARNING”,

13  ”suggestion”: “Initialize variable before first use”

14  }

15  ]

16 }

#### Workflow composition

Skills compose into end-to-end workflows. The standard ADaM development workflow chains:


(1) SK-001→SK-002→SK-003→ SK-004→SK-005→SK-006

The Agent layer orchestrates this sequence, handling dependency resolution, error recovery, and parallelization of multiple domains.

### Evaluation methodology

#### What we evaluate

Because ClinAgent is a skill and tool layer, not an LLM or agent, our evaluation validates each skill’s functional correctness: do ClinAgent’s skills and MCP tools correctly perform their specified functions? We do not evaluate how well any specific LLM reasons about clinical programming (that depends on the user’s agent choice and is outside our scope).

#### Evaluation approach: skill validation

Test types include: (i) Smoke Tests (does the skill execute without error?), (ii) Accuracy Tests (does deterministic logic produce correct outputs?), (iii) Coverage Tests (does the skill handle the full input range?), and (iv) Integration Tests (do skills correctly chain MCP tool calls?).

Metrics for deterministic components:


(2) $$\text{Detection}\;\text{Accuracy}=\frac{\text{True}\;\text{Positives}+\text{True}\;\text{Negatives}}{\text{Total}\;\text{Cases}}$$

(3) $$\text{Precision}=\frac{\text{True}\;\text{Positives}}{\text{True}\;\text{Positives}+\text{False}\;\text{Positives}}$$

For generative components:


(4) $$\text{Specification}\;\text{Match}\;\text{Rate}=\frac{\text{Variables}\;\text{correctly}\;\text{derived}}{\text{Total}\;\text{variables}\;\text{in}\;\text{specification}}$$

#### Test study selection

We evaluate on STUDY-A, using artifacts from a production Phase 2 cardiovascular study: study specifications, production SAS programs, synthetic datasets generated using the Python Faker library [19] guided by specification constraints (13 domains, 102 109 observations), and execution logs. No actual patient data was used.

#### Evaluation protocol

For each skill (SK-001 through SK-009): (i) provide skill with study artifacts, (ii) run skill’s deterministic components, (iii) record results, timing, and errors, (iv) compare outputs against ground truth, (v) classify as PASS, FAIL, or SKIP.

#### Ground truth construction

The ground truth for STUDY-A consists of four artifact classes released at the original trial’s database lock: (i) the final ADaM specifications, (ii) the production SAS programs that produced the released datasets, (iii) the SAS execution logs from the final production run, and (iv) the QC-reviewed RTF outputs delivered to the clinical study report. These artifacts were authored by the original study programming team and reviewed by an independent QC programmer following the sponsor’s standard dual-programming policy prior to release; for this validation, we treat them as the reference and did not introduce any new manual annotation pass. For the SK-005 log evaluation, the single ERROR in ADFAS and the seven WARNINGs in Table 8 were independently re-classified by the author against the SAS 9.4 Language Reference; no disagreements arose, because the rule engine matches the literal ERROR:/WARNING:/NOTE: line prefixes documented by SAS. For the SK-006 variable-validation evaluation, ground truth values were extracted programmatically from the released specification spreadsheet; only ADSL was evaluated at the variable level because it was the single domain with a fully released, machine-readable expert specification. For the SK-007 (specification generation) experiment, the reference is the expert-released ADaM specification rather than a newly annotated gold standard. We did not employ multiple independent annotators, and we did not adjudicate disagreements through consensus; the absence of an independent second annotator is acknowledged as a limitation (Limitation section). On the public CDISC Pilot01 substrate (Public Reproducibility Floor on CDISC Pilot 01 section), all oracles are frozen literals extracted from the public define.xml and ADSL dataset, requiring no expert review.

**Table 6:** Skill validation results overview

Metric	Result	Interpretation
Skills validated	9/9	All skills execute without error
Smoke test pass rate	100%	Basic functionality confirmed
Log detection precision	100%	1 Error correctly identified, 0 false positives
Variable validation accuracy	100%	56/56 ADSL variables matched specification
Code generation coverage	75%	12/16 TLF programs generated (4 skipped)

**Table 7:** Skill-by-skill validation results

ID	Skill	Scope	Status	Time (s)^a^	Validation result
SK-001	StudySetup	1 study	PASS	0.003	15 Folders created per template^b^
SK-002	SpecEditor	3 specs	PASS	0.003	56 Variables correctly parsed (STUDY-A)
SK-003	ADaMCoder	10 progs	PASS	0.002	All programs pass syntax check
SK-004	SASRunner	13 datasets	PASS	0.003	13 Datasets verified vs metadata
SK-005	LogReviewer	13 logs	PASS	2.500	1 Error detected; 0 false positives
SK-006	DataValidator	56 vars	PASS	3.500	56/56 Values match specification
SK-007	TLFCoder	16 TLFs	PASS	2.000	12 Generated, 4 skipped (no macro)
SK-008	RTFQC	20 outputs	PASS	0.004	20 RTF pairs compared^c^
SK-009	eSubPackager	5 packages	PASS	0.003	5 eSub folder structures created^d^

a The “Time (s)” column reports component-level execution of the ClinAgent code on a developer workstation (Python 3.11, single-threaded). It measures only the deterministic ClinAgent step—for example, regex scanning of a previously-written log file, structural comparison of two RTFs, or YAML/Excel parsing. It *excludes* (i) execution of the underlying SAS program (typically seconds to minutes per dataset and dependent on the SAS server), (ii) language-model inference latency for prompt-based skills, and (iii) any network or filesystem-cache effects. End-to-end wall-clock cost for a full Phase 2 study is not characterized in this work and is left to future controlled-timing studies (Limitations section).

b Template contains 14 top-level directories and 4 nested subdirectories under documents/(A&R Grid, TLF, protocol, specs) for 18 total nodes (R1 oracle on Pilot01: 18 nodes).

c Pilot01 substrate has two RTF pairs (tlf-primary.rtf versus tlf-efficacy.rtf); R8 oracle confirms non-match with production_length = 723, qc_length = 304.

d Each eSub skeleton template contains 12 directory/file entries (R9 oracle on Pilot01: 12 entries per package).

**Table 8:** SAS Log analysis results (SK-005)

Domain	Status	Errors	Warnings	Notes
ADSL	CLEAN	0	0	1372
ADAE	WARNING	0	3	2454
ADBASE	CLEAN	0	0	1235
ADCM	CLEAN	0	0	845
ADDILI	CLEAN	0	0	1372
ADEFF	WARNING	0	2	1254
ADEG	WARNING	0	1	1683
ADEX	CLEAN	0	0	1161
ADFAS	**ERROR**	**1**	1	108
ADLB	CLEAN	0	0	1629
ADMH	CLEAN	0	0	512
ADSOC	CLEAN	0	0	1234
ADVS	CLEAN	0	0	1335
**Total**		**1**	**7**	**16 194**

Bold values highlight the specific error detected and the overall column totals.

#### Reproducibility

Software environment: Python 3.11+ with pyreadstat (SAS7BDAT reading), openpyxl (Excel parsing), pandas (data manipulation); MCP SDK version 1.0+ for tool server implementation. Skills are defined as Markdown files with YAML frontmatter. Architecture specifications, MCP server source, and skill definitions are available in the Supplementary Material and at https://github.com/yanmingyu92/ClinAgent.

To clarify which components a reader can reproduce without proprietary software, we partition the repository as follows. Reproducible without SAS: all six MCP tools (sas-dataset-reader reads via the open pyreadstat library; excel-spec-editor, sas-log-analyzer, macro-catalog, rtf-comparator, plus filesystem), all rule engines (SK-001, SK-002, SK-005, SK-006, SK-008, SK-009), synthetic-dataset generation, and the entire CDISC Pilot01 test suite (pytest bench-program/track1-pilot01 -q on a cold clone; requiring only Python 3.11+ and open-source packages, with no SAS license). Requires a licensed SAS 9.4 installation: SK-004 (SASRunner) end-to-end execution and the SAS-execution portion of SK-003/SK-007. Code generation for SK-003 and SK-007 is reproducible without SAS; only program *execution* requires a licensed SAS environment. Requires an MCP-capable language-model agent: prompt-based specification generation (Results section, Table 9); ClinAgent itself contains no language model, so the agent and its API key are user-supplied. A docker compose up workflow and a Pilot01-only quick-start are documented in the repository README.md.

**Table 9:** Specification generation accuracy by domain^a^

Domain	Vars Match	Extra	Missing	Deriv. acc. (%)
ADMH	17	0	2	**100.0**
ADEX	30	4	0	96.7
ADCM	52	9	0	96.2
ADVS	33	7	3	69.7
ADEG	41	5	9	70.7
ADAE	75	46	13	65.3
ADLB	49	22	11	59.2
ADSL	46	53	10	54.3
ADBASE	5	0	66	0.0
**Overall**	**348**	**146**	**114**	**72.1**

a Derivation accuracy is computed as the fraction of expert-defined derived variables correctly captured by the AI-generated specification.

Bold values highlight the highest achieved derivation accuracy and the overall totals across all evaluated domains.

#### Limitations of this evaluation

We acknowledge: (i) single-study, proof-of-concept scope (broader generalization requires multi-study replication); (ii) tool validation does not equal end-to-end submission validation; (iii) no controlled timing study at the wall-clock level; (iv) ground-truth dependency on artifacts authored by the original study team without an additional independent annotator for this article. These limitations define the scope for future work and are revisited in the Limitation section.

## Results

### Validation study overview

We validated ClinAgent’s nine skills using artifacts from STUDY-A, a production Phase 2 cardiovascular/lipids study. This validation study used study specifications, production SAS programs, and synthetic datasets; no actual patient data were used. These artifacts provide ground truth (expert-written programs, reviewed logs, specification-compliant outputs) for measuring skill correctness. This is a single-study proof-of-concept validation: results characterize the ClinAgent tooling on one Phase 2 cardiovascular study and should not be read as evidence of multi-study, cross-therapeutic-area, or production-scale performance. Broader generalizability requires the multi-study replication outlined in Limitation section.

Results in this section reflect two evaluation modes that the reader should keep distinct. (i) Deterministic-tool validation (Log Analysis Results, Specification Generation Accuracy, TLF Generation Results, Dataset Characteristics sections, Tables 6–12) measures the correctness of rule engines and parsers that run without any language model in the loop; the perfect point-estimate figures reported below apply only to mode (i) and are interpreted with Wilson confidence intervals. (ii) Prompt-dependent specification generation (Specification Generation Accuracy section, Table 9 and Fig. 5) measures the output of a language-model agent guided by a ClinAgent skill prompt; those results depend on the underlying model and are reported separately. Wall-clock execution timings in tables throughout this section refer to component-level execution of the deterministic ClinAgent code on a developer workstation and exclude SAS execution time and language-model inference latency.

**Table 10:** ADaM dataset characteristics (STUDY-A)

Domain	Obs.	Vars.	Description
ADSL	669	56	Subject-level analysis
ADAE	260	109	Adverse events
ADBASE	381	72	Baseline characteristics
ADCM	3210	52	Concomitant medications
ADDILI	4863	62	Drug-induced liver injury
ADEFF	16 831	65	Efficacy endpoints
ADEG	5697	71	ECG analysis
ADEX	2144	51	Exposure
ADFAS	1888	24	Fasting status
ADLB	55 391	81	Laboratory results
ADMH	1540	19	Medical history
ADSOC	4120	45	System organ class
ADVS	5115	57	Vital signs
**Total**	**102 109**	**764**	(STUDY-A; proprietary synthetic data)

Bold values highlight the overall totals for observations and variables across all evaluated domains.

**Table 11:** Key metrics with 95% Wilson score confidence intervals

Metric	*n*	Successes	Point est. (%)	95% CI (Wilson)
Skill pass rate	9	9	100.0	70.1–100.0
Log error detection (precision)	1	1	100.0	20.7–100.0
Log warning detection (precision)	7	7	100.0	64.6–100.0
Variable validation accuracy	56	56	100.0	93.6–100.0
TLF generation success	16	12	75.0	50.5–89.8
Overall derivation accuracy	348	251	72.1	67.2–76.6

**Table 12:** Log analysis confusion matrix (SK-005)

	Predicted		
Actual	Positive (Issue)	Negative (clean)	Total
Positive (issue)	8 (TP)	0 (FN)	8
Negative (clean)	0 (FP)	16 186 (TN)	16 186
**Total**	**8**	**16 186**	**16 194**

Bold values highlight the overall row and column totals. TP = True Positive; TN = True Negative; FP = False Positive; FN = False Negative.

**Figure 3:**
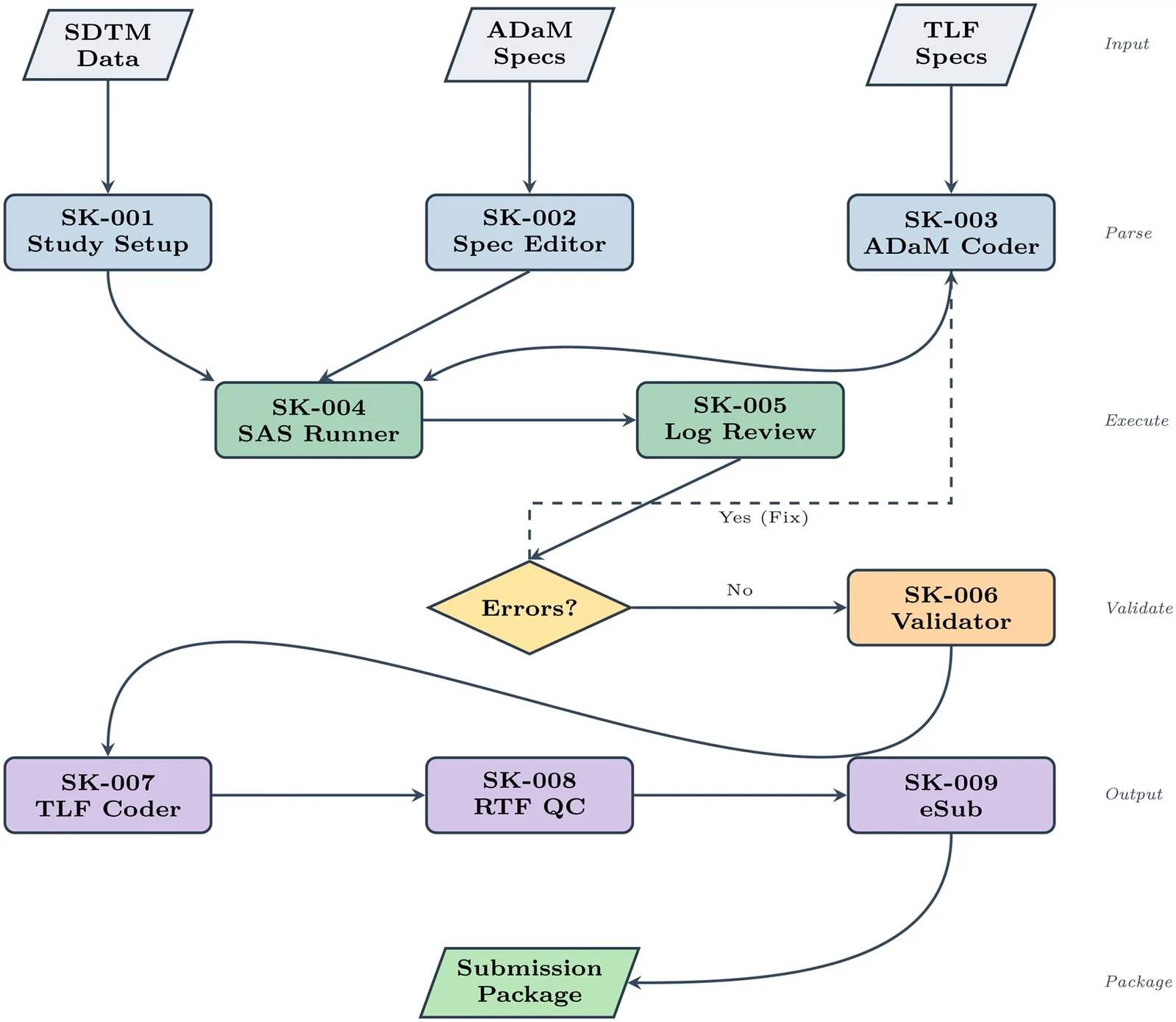
ClinAgent end-to-end workflow. The framework orchestrates 9 skills through the complete clinical trial programming pipeline from SDTM data to regulatory submission package. Input data (SDTM, ADaM specs, TLF specs) flows through parsing (SK-001, SK-002, SK-003), execution (SK-004, SK-005), validation (SK-006), output processing (SK-007, SK-008, SK-009), and final packaging. Dashed arrow indicates the error recovery loop.

Figure 3 illustrates the end-to-end workflow validated in this study.

### Test study characteristics

Table 5 summarizes the artifact characteristics of STUDY-A.

### Skill validation summary

Table 6 summarizes validation results. These metrics measure tool correctness, not LLM performance.

### Detailed skill validation results

**Figure 4:**
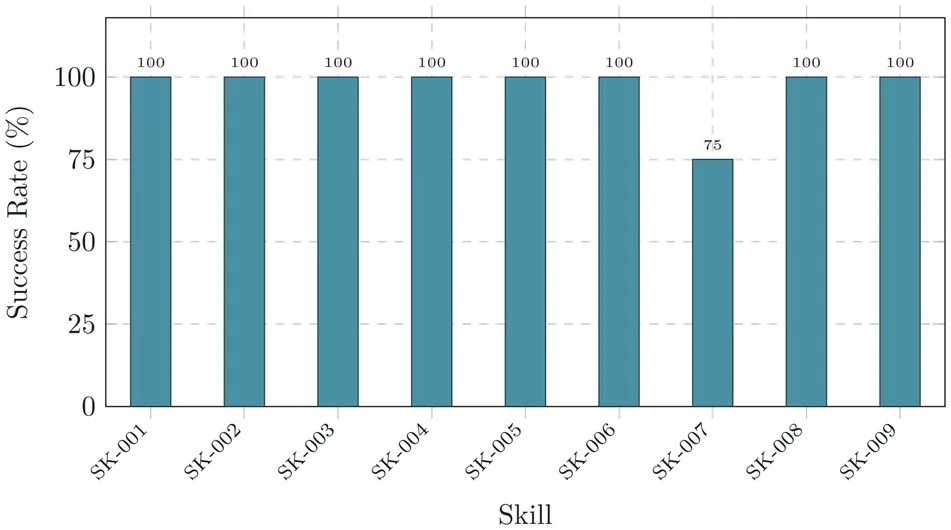
Skill success rates on STUDY-A validation tests. All nine skills achieved 100% success except SK-007 (TLF coder) at 75% due to four TLFs lacking required macro specifications. Success rates reflect deterministic skill component correctness, not LLM reasoning performance.

Table 7 presents validation results for each skill, and Fig. 4 shows the corresponding per-skill success rates.

### Log analysis results

Table 8 presents batch log analysis across all 13 ADaM domains.

In this validation sample, the log reviewer correctly identified the single error in ADFAS (“ERROR: Cannot open WORK.FAPLUS.DATA for output access”) with no false positives (1 true positive, 0 false positives). The confidence interval implications of this small event count are reported in Log Analysis Classification Performance section and Table 11.

### Specification generation accuracy

Table 9 shows accuracy of AI-generated specifications compared to expert reference. Specification generation was evaluated on the nine domains with available expert-written reference specifications; the remaining four domains (ADFAS, ADDILI, ADEFF, ADSOC) were used only for tool validation and log analysis.

**Figure 5:**
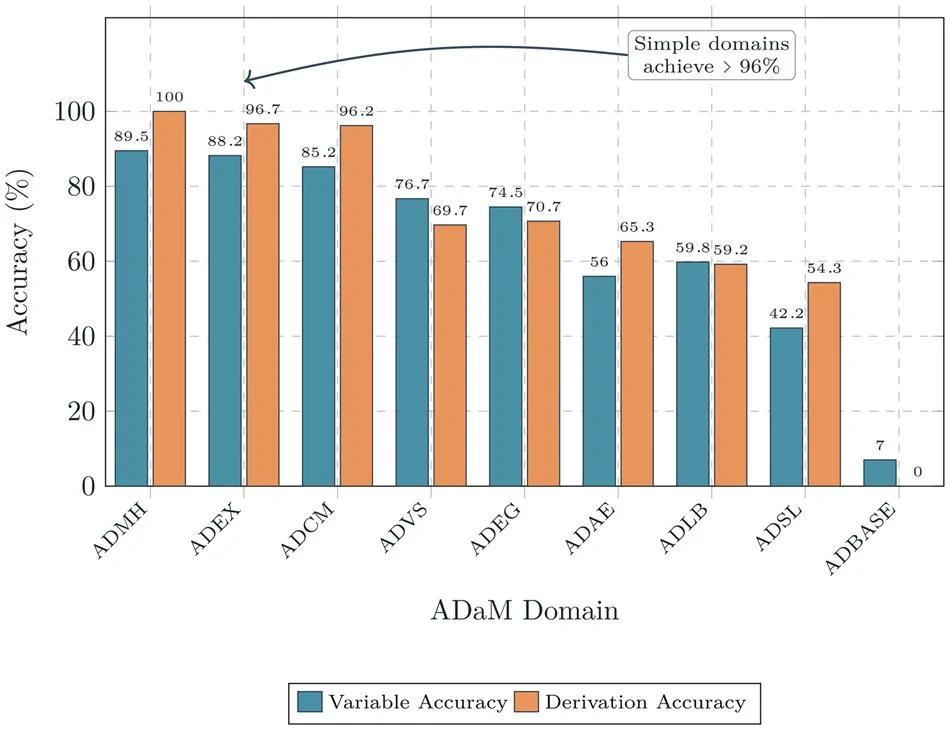
Specification generation accuracy varies by domain complexity. Simple domains (ADMH, ADEX, ADCM) achieve >85% variable accuracy and >96% derivation accuracy. Complex domains with many study-specific derived variables (ADSL, ADBASE) show significantly lower accuracy. Blue bars show variable accuracy; orange bars show derivation accuracy.

Simple domains (ADMH, ADEX, ADCM) achieve >96% derivation accuracy. Complex domains with many derived variables (ADSL, ADBASE) show lower accuracy (Fig. 5).

Implication for human review. Given an overall derivation accuracy of 72.1% and a 0% result on ADBASE, AI-generated ADaM specifications cannot be treated as ready-for-coding artifacts in this proof-of-concept setting. Any operational use should treat the generated specification as a *draft* that requires full expert review before it is consumed by downstream skills (SK-003 ADaMCoder, SK-004 SASRunner). Based on the per-domain pattern in Table 9, we recommend at minimum: (i) variable-by-variable expert review for any domain in which more than 30% of variables are derived rather than copied from SDTM; (ii) mandatory dual review (programmer + statistician sign-off) for ADSL- and ADBASE-class subject-level/baseline domains, where derivation logic is most study-specific; (iii) automated diff of the generated specification against a prior released specification when one exists, with manual adjudication of every removed or added derivation; and (iv) explicit rejection of generated content for any domain whose derivation accuracy falls below a sponsor-defined threshold (e.g. 90%). These recommendations reflect the validation evidence in this article rather than a regulatory requirement.

### TLF generation results

The TLF Coder (SK-007) generated 12 programs from 16 TLF specifications. Four programs were skipped because specifications lacked required macro names. This is a data quality issue, not a skill failure. All generated programs include required components (standard header, log redirection, data preparation, macro call, log checker invocation, wrapup procedures).

### Dataset characteristics

Table 10 summarizes the ADaM datasets processed in this validation study.

### Statistical analysis

#### Confidence intervals

For proportions based on small samples, the Wilson score interval [20] [Equation (5)] provides better coverage:


(5) $$\hat{p}\pm \frac{z_{\alpha /2}}{1+\frac{z_{\alpha /2}^{2}}{n}}\sqrt{\frac{\hat{p}(1-\hat{p})}{n}+\frac{z_{\alpha /2}^{2}}{4n^{2}}}$$

where $\hat{p}$ represents the point estimate of the success proportion, *n* denotes the sample size, and $z_{\alpha /2}$ is the quantile of the standard normal distribution corresponding to the target significance level (e.g. 1.96 for a 95% confidence interval).

Table 11 reports 95% Wilson CIs for key metrics.

#### Log analysis classification performance

Ground truth for log classification was established from the production log-review artifacts described in Ground Truth Construction section, with author re-classification of the one ERROR and seven WARNINGs against the SAS Language Reference. Table 12 presents the confusion matrix.

On this sample, sensitivity, specificity, precision, negative predictive value, and F1 score all reached 100%, with Matthews Correlation Coefficient (MCC) = 1.000 providing a balanced measure under the extreme class imbalance (8 issues versus 16,186 clean lines). From a machine-learning perspective, these scores would normally indicate a classifier that perfectly reproduces the expert label set, and on this validation sample, that is what we observed. However, the underlying mechanism is *not* a learned model: SK-005 is a deterministic regex rule engine that classifies lines by matching the literal ERROR:/WARNING:/NOTE: prefixes documented in the SAS Language Reference and by suppressing a small curated list of known-benign warnings (e.g. Unable to copy SASUSER). The contribution of ClinAgent at this layer is therefore *rule curation*—identifying which line patterns expert reviewers treat as actionable versus benign—not learning behavior. The 100% alignment indicates that the curated rule set covers all issue patterns present in this study’s 13 logs; it does not imply that the rule set will generalize to logs containing patterns that have not yet been curated, nor to studies whose programming teams emit warnings or notes not represented in this sample.

Caution on small-sample confidence intervals. The 100% scores rest on only 8 true positives and 0 false negatives across the 13 production logs. As reported in Table 11, the corresponding 95% Wilson interval for log error detection precision is [20.7–100%] (1/1 errors) and for warning detection precision is [64.6–100%] (7/7 warnings). These intervals are wide because the event counts are small, not because the rule engine performs poorly; we present them transparently and discourage interpreting the 100% point estimates as evidence of production-scale generalization. Multi-study replication on logs with greater issue density—and adversarial curation of warning patterns not yet seen—is required before any operational claim of regulatory-grade log review can be made.

#### Correlation analysis: domain complexity versus accuracy

Spearman’s rank correlation between complexity metrics and derivation accuracy:

- Total Variables versus Accuracy: ρ=−0.683, P=.042
- Derived Variables versus Accuracy: ρ=−0.817, P=.007
- Proportion Derived versus Accuracy: ρ=−0.78, P=.012

**Figure 6:**
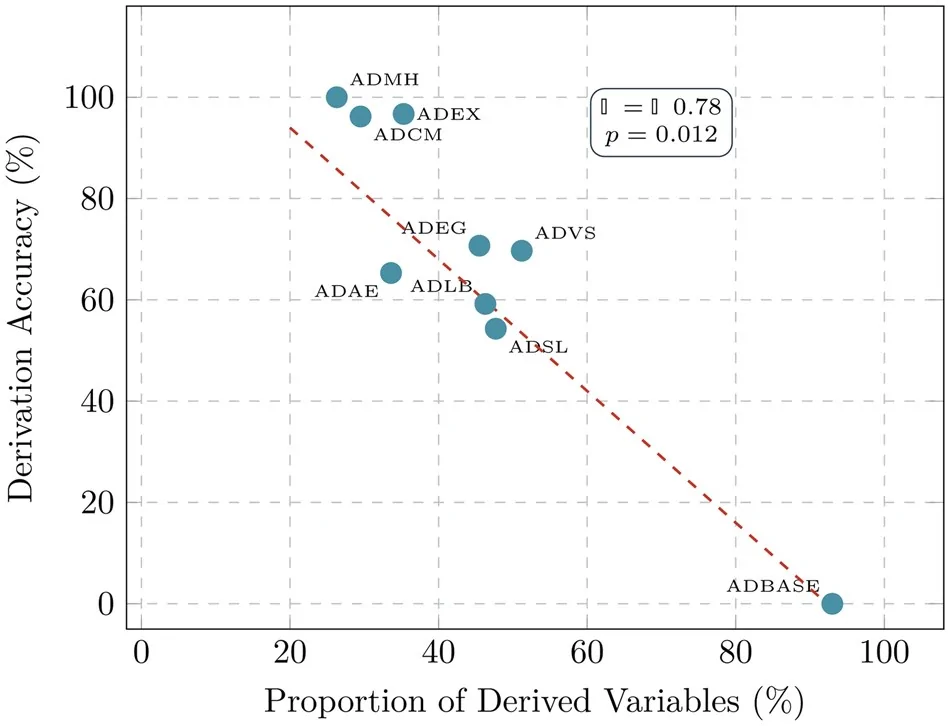
Correlation between domain complexity (proportion of derived variables) and specification generation accuracy. Strong negative correlation (ρ=−0.78, P=.012) indicates AI-generated specifications are significantly less accurate for domains with more study-specific derived variables. The dashed line is a visual guide, not a fitted regression line.

There is a statistically significant negative correlation between the proportion of derived variables and specification accuracy (ρ=−0.78, P=.012), as shown in Fig. 6.

#### Effect size analysis

We computed Cohen’s *d* between the simple-domain group (ADMH, ADEX, ADCM; group size $n_{s}=3$, mean derivation accuracy ${\bar{x}}_{\text{simple}}=97.6\%$, standard deviation $s_{s}=2.1\%$) and the complex-domain group (ADVS, ADEG, ADAE, ADLB, ADSL, ADBASE; group size $n_{c}=6$, mean derivation accuracy ${\bar{x}}_{\text{complex}}=53.2\%$, standard deviation $s_{c}=26.8\%$):


(6) $$\text{Cohe}n^{\prime }s\;d=\frac{{\bar{x}}_{\text{simple}}-{\bar{x}}_{\text{complex}}}{s_{\text{pooled}}}=\frac{97.6-53.2}{22.1}=2.01,$$

where the pooled standard deviation is


$$s_{\text{pooled}}=\sqrt{\frac{(n_{s}-1)s_{s}^{2}+(n_{c}-1)s_{c}^{2}}{n_{s}+n_{c}-2}}=\sqrt{\frac{2\cdot 2.1^{2}+5\cdot 26.8^{2}}{7}}\approx 22.1\%.$$

${\bar{x}}_{\text{simple}}$ and ${\bar{x}}_{\text{complex}}$ are the per-domain derivation accuracies (Table 9) averaged within each group, and $s_{s}$, $s_{c}$ are the corresponding within-group sample standard deviations. The resulting d=2.01 exceeds the conventional “large” threshold of 0.8 (Cohen’s small/medium/large landmarks: 0.2/0.5/0.8) and confirms that the performance difference between simple and complex domains is practically, not just statistically, substantial. We note that with only $n_{s}+n_{c}=9$ domains the effect-size estimate itself has wide uncertainty; this analysis is descriptive of STUDY-A and not a generalization claim.

### Public reproducibility floor on CDISC Pilot01

To enable independent verification of ClinAgent’s deterministic components, we executed six of the nine skills on the publicly available CDISC Pilot01 study [21] from the eSubmission-Benchmark repository (commit 658fcc05, CC-BY-4.0 license). Pilot01 is an external substrate and does not share data with STUDY-A; it provides a *reproducibility floor*, not a re-evaluation of STUDY-A results. The three skills excluded (SK-003 ADaMCoder, SK-004 SASRunner, SK-007 TLFCoder) require study-specific LLM prompting or a local SAS installation; the remaining six skills exercise deterministic tooling only and are fully reproducible from a cold clone.

All oracles are frozen literals tested against the CDISC Pilot01 substrate. The full test suite (15 tests including 2 integrity checks) is runnable via pytest bench-program/track1-pilot01 -q on a cold clone of the repository using Python 3.11+ and open-source packages only; no SAS license is required for the Pilot01 reproducibility path. Table 13 reports only the 13 skill-specific tests. As with Table 7, the “Time (s)” column reports component-level execution of the ClinAgent deterministic code (regex scan, structural diff, define.xml parse) on a developer workstation; it excludes any underlying SAS execution and language-model inference. The sub-second figures should not be read as end-to-end pipeline timings.

**Table 13:** Skill execution results on CDISC Pilot01 (public substrate)

Test	Skill	Oracle	Result	Time (s)
R1	SK-001 StudySetup	Template yields 18 nodes	PASS	<0.01
R2	SK-002 SpecEditor	ADSL define.xml: 5 datasets, 231 ItemDefs	PASS	<0.01
R3	SK-006 DataValidator	ADSL ITTFL = ‘Y’ =254	PASS	0.02
R4	SK-006 DataValidator	TRT01PN: Placebo =86, Low =84, High =84	PASS	0.02
R5	SK-006 DataValidator	SAFFL ≤ ITTFL; EFFFL ≤ ITTFL	PASS	0.02
R6	SK-006 DataValidator	Placebo mean age =75.21	PASS	0.02
R7	SK-008 RTFQC	RTF self-compare: match=true	PASS	<0.01
R8	SK-008 RTFQC	RTF cross-compare: match=false	PASS	<0.01
R9	SK-009 eSubPackager	Skeleton: 12 entries per package	PASS	<0.01
R10	SK-005 LogReviewer	Error log: 1 ERROR detected	PASS	0.01
R11	SK-005 LogReviewer	Warning log: 1 WARNING detected	PASS	0.01
R12	SK-005 LogReviewer	Clean log: 0 errors, 0 warnings	PASS	0.01
P1	SK-008 Parity	TypeScript ≡ Python mirror	PASS	0.21
**Total**			**13/13**	**0.82**

Bold values highlight the overall totals for the execution results and time.

Four evaluation dimensions are exercised on Pilot01, mirroring the STUDY-A protocol (Results section):

1. Log detection precision (SK-005): three seeded SAS programs—clean, warning, and error—were executed on a licensed SAS 9.4 instance. The log reviewer correctly identified the single error (Libref NOSUCH is not assigned) and single warning (Multiple lengths specified for variable x), with zero false positives on the clean run (precision =100%; 3/3 correct classifications).
2. Data validation accuracy (SK-006): four ADSL population metrics—ITT count, treatment-group sizes, flag-subset invariants, and placebo age mean—all match their frozen oracles extracted from the Pilot01 ADSL dataset (accuracy =100%; 4/4 metrics).
3. Structural parsing (SK-002): the Pilot01 define.xml is correctly parsed into 5 ADaM datasets and 231 ItemDef entries, confirming specification-reader correctness on a real CDISC artifact.
4. Cross-language concordance (SK-008): the TypeScript MCP server and its Python mirror produce identical match and length fields on both the self-compare (R7) and cross-compare (R8) tests, confirming inter-implementation agreement.

## Discussion

### Key findings

#### Agent-Augmentation architecture works

Our validation demonstrates that the agent-augmentation model (providing domain tools to existing AI agents rather than building a new agent) is viable: (i) all nine skills passed validation, confirming deterministic rule engines and parsers produce correct outputs; (ii) skills successfully expose functionality via MCP, enabling any compatible agent to use ClinAgent tools; (iii) the “Thin MCP, Thick Skills” principle keeps data access simple while domain logic remains testable.

#### Deterministic components excel

Skills with rule-based components achieved high accuracy on STUDY-A: SK-005 Log Reviewer identified the observed error and warnings with no false positives, and SK-006 Data Validator matched all 56 evaluated ADSL variables. The corresponding 95% Wilson intervals (Table 11) are wide—[20.7–100%] for the single-error precision and [64.6–100%] for warning precision—so these point estimates should be read as evidence that deterministic rules can in principle handle classification and validation tasks correctly on this study, not as proof of production-scale performance.

#### Generative components show domain variation

AI-generated specifications showed variable accuracy: simple domains ADMH (100%), ADEX (96.7%), ADCM (96.2%) versus complex domains ADVS (69.7%), ADSL (54.3%), ADBASE (0%). Overall 72.1% derivation accuracy indicates that, in this proof-of-concept setting, AI-generated specifications are useful drafts but are not ready for downstream consumption without expert review; the level of review we recommend is detailed at the end of Results section.

### Advantage over existing approaches

ClinAgent offers several advantages over existing approaches to clinical programming automation. Relative to manual programming and generic AI agents without domain tools, ClinAgent’s deterministic validation tools provide systematic, auditable coverage of log review and data-validation tasks, while augmented agents can perform clinical-data operations that are otherwise impossible or unreliable without domain-specific readers and rule engines. Furthermore, while template-based or rule-macro approaches are rigid, ClinAgent provides AI-agent integration via standard protocols and end-to-end workflow orchestration. Building a custom clinical programming agent would require substantial effort; in contrast, ClinAgent’s augmentation approach offers lower development cost, automatic benefit from agent improvements, user flexibility in agent choice, and easier enterprise adoption. The “Thin MCP, Thick Skills” design ensures that new capabilities are primarily configuration (adding rules to JSON files) rather than code changes, enabling domain experts to extend ClinAgent without programming knowledge.

### Generalization to biology and medicine research pipelines

Although ClinAgent was designed for clinical trial statistical programming, its architecture generalizes to other biology and medicine research pipelines. The core insight, that AI agents need domain-specific tool layers to be productive in specialized workflows, applies across biomedical research.

In observational studies and epidemiological research, data processing pipelines share structural similarities with clinical trial workflows: raw data must be transformed, validated, and formatted for analysis according to domain-specific standards. ClinAgent’s skill pattern (embedded prompts + rule engines + tool bindings) could be adapted for epidemiological data harmonization across disparate health registries.

In real-world data (RWD) analytics, where electronic health records, claims databases, and patient registries require similar extract-transform-load processes, ClinAgent’s architecture could provide standardized tool layers for common data models (OMOP CDM, Sentinel) that AI agents currently cannot access natively.

In bioinformatics pipelines, where sequence data must be processed through standardized workflows (quality control, alignment, variant calling, annotation), the same “Thin Tools, Thick Skills” pattern applies: AI agents need domain-specific tools for file format access (FASTQ, BAM, VCF) and knowledge packaging (reference genome annotations, variant databases).

In each case, the key architectural principle remains: separate deterministic data access and validation (tools) from domain reasoning and workflow logic (skills), while allowing the user’s preferred AI agent to handle general reasoning. This separation of concerns enables domain experts to build and maintain skill libraries without modifying the underlying tool infrastructure.

### Ethical and regulatory considerations

#### Data privacy

ClinAgent addresses PHI protection through: local processing (no patient data transmitted to external AI services unless explicitly configured), DataMasker component for PHI/PII removal, context minimization, and audit logging of all data access.

#### Regulatory compliance

ClinAgent’s design includes features commonly required by FDA 21 CFR Part 11 and EU Annex 11 [22]: deterministic rule engines that produce reproducible outputs from the same inputs, a complete audit log of every tool invocation, an explicit separation between validated deterministic tools and stochastic language-model reasoning, and the structural assumption that a human remains in the loop for any safety-relevant decision. These are *design enablers*, not a compliance certification. ClinAgent itself has not undergone formal computer-system validation (CSV) or computer-software-assurance (CSA) qualification within any specific quality management system; we make no claim of 21 CFR Part 11 or Annex 11 compliance for the released codebase. Audit logging, PHI masking, and deterministic rule execution reduce the risk surface but do not, on their own, establish regulatory compliance: that determination must be made by each adopting organization against its own validation policy, risk classification, intended use, and electronic-records/electronic-signatures controls.

### Limitations

Our evaluation is a single-study, proof-of-concept validation of deterministic tool correctness; we have not validated end-to-end productivity gains, generalization across therapeutic areas or trial phases, or performance with different language-model agents. The scope of this article is the *software and functional capabilities* of the ClinAgent skill and tool layer (see Contribution 4 in Introduction and Evaluation Methodology sections); cross-agent benchmarking of the underlying language models that invoke ClinAgent is intentionally out of scope. Cross-validation on multiple studies across different therapeutic areas, with the specifications and logs of each study acting as independent ground truth, is required before generalizability can be claimed. Accuracy metrics depend on expert-authored ground-truth artifacts: for STUDY-A, only one annotation pass (the original study team’s dual-programming review) is available, and the present article did not introduce an additional independent annotator (Ground Truth Construction section). Results assume well-structured input specifications; real-world specifications vary in completeness, and degraded inputs are expected to lower accuracy. Statistical power for log detection metrics is limited by the small number of true issues (eight total) and the corresponding 95% Wilson intervals are correspondingly wide (Table 11). Finally, the regulatory-compliance features described in Regulatory Compliance section are design enablers and are not equivalent to formal CSV/CSA qualification.

### Future work

Priority extensions include: (i) multi-study validation across therapeutic areas and phases, (ii) controlled productivity studies measuring end-to-end time savings, (iii) skill expansion to SDTM mapping, define.xml generation, and Pinnacle 21 compliance, (iv) regulatory engagement on AI tool validation pathways, and (v) adaptation of the architecture to observational research and real-world data pipelines as discussed in Section 4.3.

## Supplementary Material

bpag032_Supplementary_Data

## Data Availability

Source code: https://github.com/yanmingyu92/ClinAgent (commit hash pinned in the supplement). Synthetic evaluation datasets are derived from the public CDISC pilot project. A Zenodo DOI for the release snapshot will be issued at acceptance.

## Preprint

A previous version of this manuscript was posted on medRxiv (doi: 10.64898/2026.01.09.26343542).
