# Supplementary material for: ClinAgent: AI-assisted methodology for clinical trial data processing and statistical programming
Source: Biol Methods Protoc. 2026 Jun 11;11(1):bpag032. doi: 10.1093/biomethods/bpag032 (PMC13555824; doi:10.1093/biomethods/bpag032)
Supplement: Supplementary file 1 [file bpag032_supplementary_data.zip › supplementary_bmp.pdf]

Supplementary Material

ClinAgent: AI-Assisted Methodology for Clinical Trial Data Processing and Statistical Programming

Submitted to *Biology Methods & Protocols*

Jaime Yan

PhD Program, Harrisburg University of Science and Technology, Harrisburg, PA, USA

ORCID: 0009-0007-1786-7259

This document accompanies the main manuscript submitted to *Biology Methods & Protocols*. It provides the complete skill catalogue (Appendix A) referenced from the Methods section. All numerical results, figures, and tables in the main manuscript are derived from the artefacts described here.

A Skill Definitions

A.1 Complete Skill Specifications

SK-001: Study Setup

- **Purpose**: Create standardized folder structure for new study
- **Inputs**: study_id, protocol_number, therapeutic_area
- **Outputs**: Folder hierarchy, template files, configuration
- **Rules**: Naming conventions, required subfolders

SK-002: Specification Editor

- **Purpose**: Parse and edit ADaM specifications
- **Inputs**: Excel specification file, edit instructions
- **Outputs**: Updated specification, change log
- **Rules**: Column validation, controlled terminology

SK-003: ADaM Coder

- **Purpose**: Generate ADaM SAS programs from specifications
- **Inputs**: Specification, SDTM metadata, macro catalog
- **Outputs**: SAS program file, documentation
- **Rules**: Macro selection, variable derivation patterns

SK-004: SAS Runner

- **Purpose**: Execute SAS programs in batch
- **Inputs**: Program path, execution parameters
- **Outputs**: Execution status, log file path, dataset paths
- **Rules**: Environment setup, error handling

SK-005: Log Reviewer

- **Purpose**: Analyze SAS logs for issues
- **Inputs**: Log file path, severity threshold
- **Outputs**: Issue list, severity classification, recommendations
- **Rules**: 50+ error patterns, severity classification

SK-006: Data Validator

- **Purpose**: Validate datasets against specifications
- **Inputs**: Dataset path, specification, validation rules
- **Outputs**: Validation report, discrepancy list
- **Rules**: Type checking, length validation, controlled terms

SK-007: TLF Coder

- **Purpose**: Generate TLF programs from shells
- **Inputs**: TLF shell, ADaM datasets, macro catalog
- **Outputs**: SAS program, mock output
- **Rules**: Macro selection, formatting standards

SK-008: RTF QC

- **Purpose**: Compare RTF outputs for QC

- **Inputs**: Production RTF, QC RTF, tolerance settings
- **Outputs**: Comparison report, difference highlights
- **Rules**: Numeric tolerance, whitespace handling

SK-009: eSub Packager

- **Purpose**: Create regulatory submission package
- **Inputs**: Study artifacts, submission type
- **Outputs**: eSub folder structure, define.xml
- **Rules**: FDA/EMA requirements, file naming

A.2 Rule Engine Schema

Listing 1: Rule Engine JSON Schema

```
{
  "$schema": "http://json-schema.org/draft-07/schema#",
  "type": "object",
  "properties": {
    "rule_id": {"type": "string"},
    "name": {"type": "string"},
    "description": {"type": "string"},
    "condition": {
      "type": "object",
      "properties": {
        "field": {"type": "string"},
        "operator": {"enum": ["eq", "ne", "gt", "lt", "regex", "in"]},
        "value": {}
      }
    },
    "action": {
      "type": "object",
      "properties": {
        "type": {"enum": ["classify", "transform", "validate", "alert"]},
        "parameters": {"type": "object"}
      }
    },
    "priority": {"type": "integer", "minimum": 1, "maximum": 100}
  },
```

```
  "required": ["rule_id", "name", "condition", "action"]
}
```
